# Supplementary material for: Effect of CaCO3 Particle Size on Surface Wetting and Adhesion: Studies on PMMA Model Substrates and Laurus nobilis Leaves
Source: Plants (Basel). 2025 Dec 17;14(24):3838. doi: 10.3390/plants14243838 (PMC12737221; doi:10.3390/plants14243838)
Supplement: Supplementary file 1 [file plants-14-03838-s001.zip › plants-3961364-supplementary.pdf]

Supplementary Materials

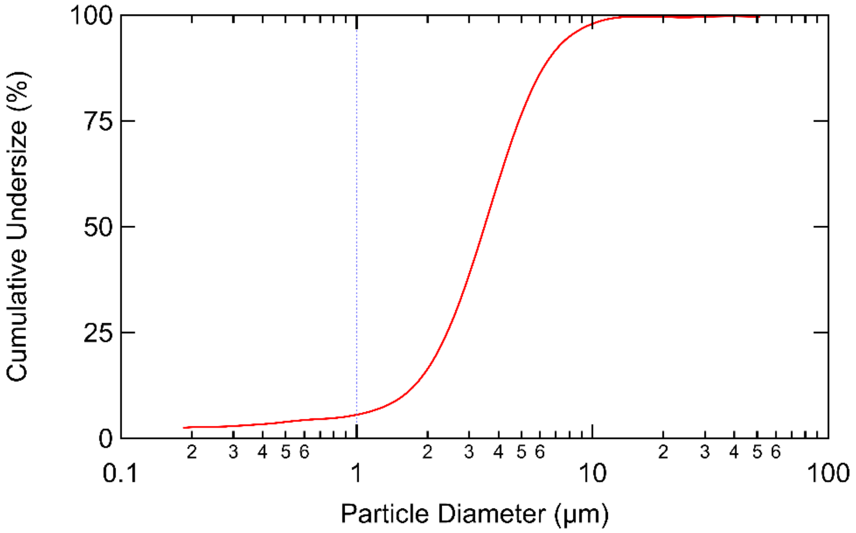

Figure S1. Particle size distribution (PSD) of microparticles determined by sedimentation technique and Stockes law. The red curve represents the volume-based cumulative undersize percentage. The data indicate a narrow size range, with a volume-based d50 of 3.5 µm and a d98 of 9.9 µm.

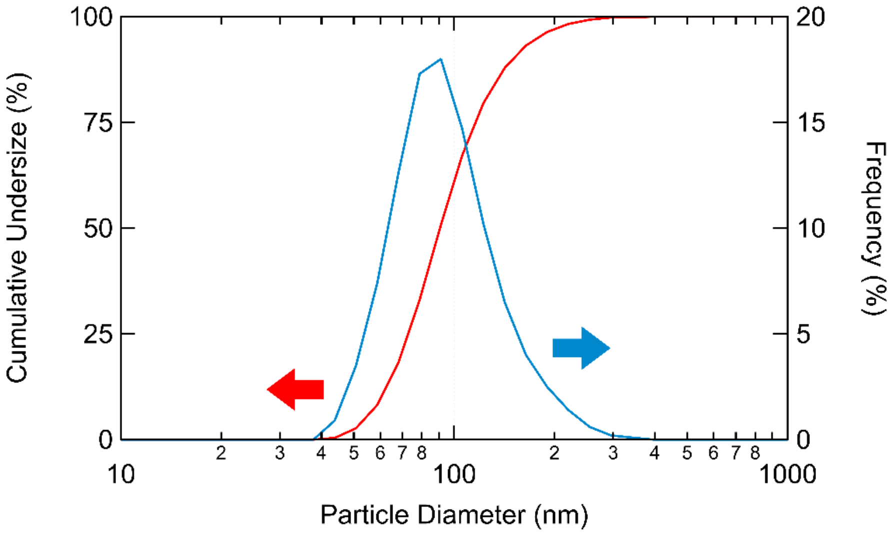

Figure S2. Particle size distribution (PSD) of synthesized nanoparticles determined by dynamic light scattering (DLS). The blue curve represents the number-based frequency distribution of particle diameters (right axis), while the red curve shows the number-based cumulative undersize percentage (left axis). The data indicate a narrow size range, with a number-based d50 of 91 nm and a d98 of 214 nm, confirming good homogeneity of the nanoparticle population.

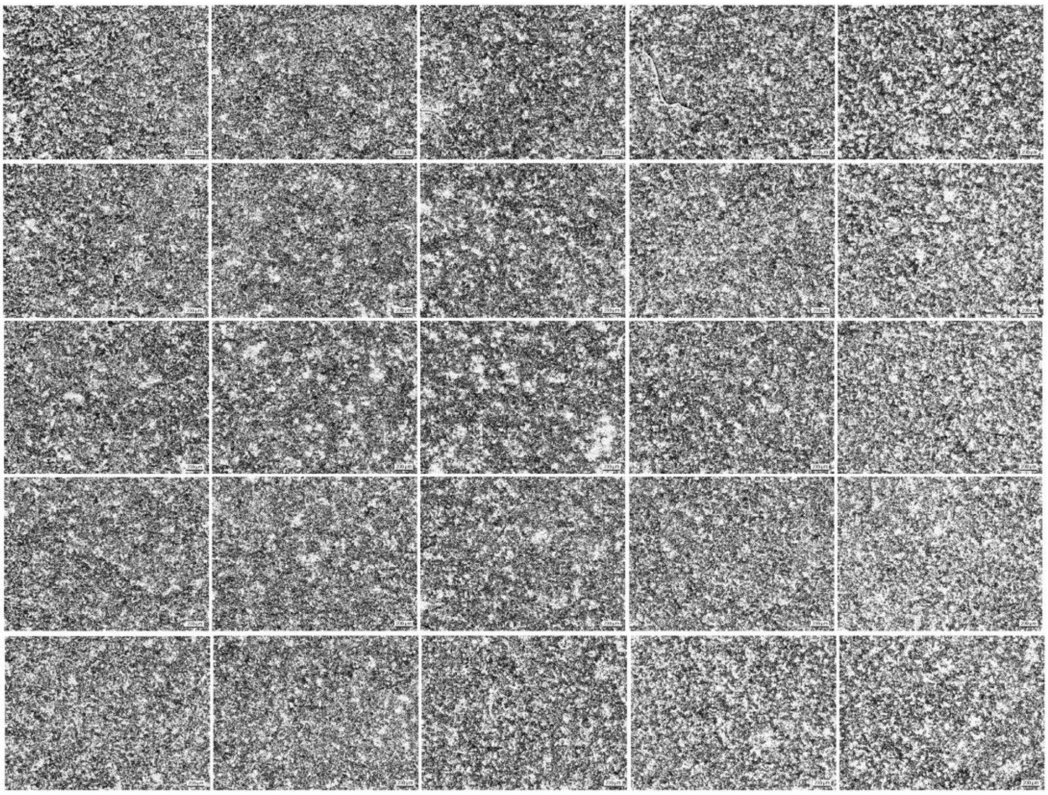

Figure S3. Array of SEM micrographs showing the PMMA plate coated with microparticles (MP_surf) prior to rinsing. These images were used to calculate the coating coverage values presented in Figure 4.

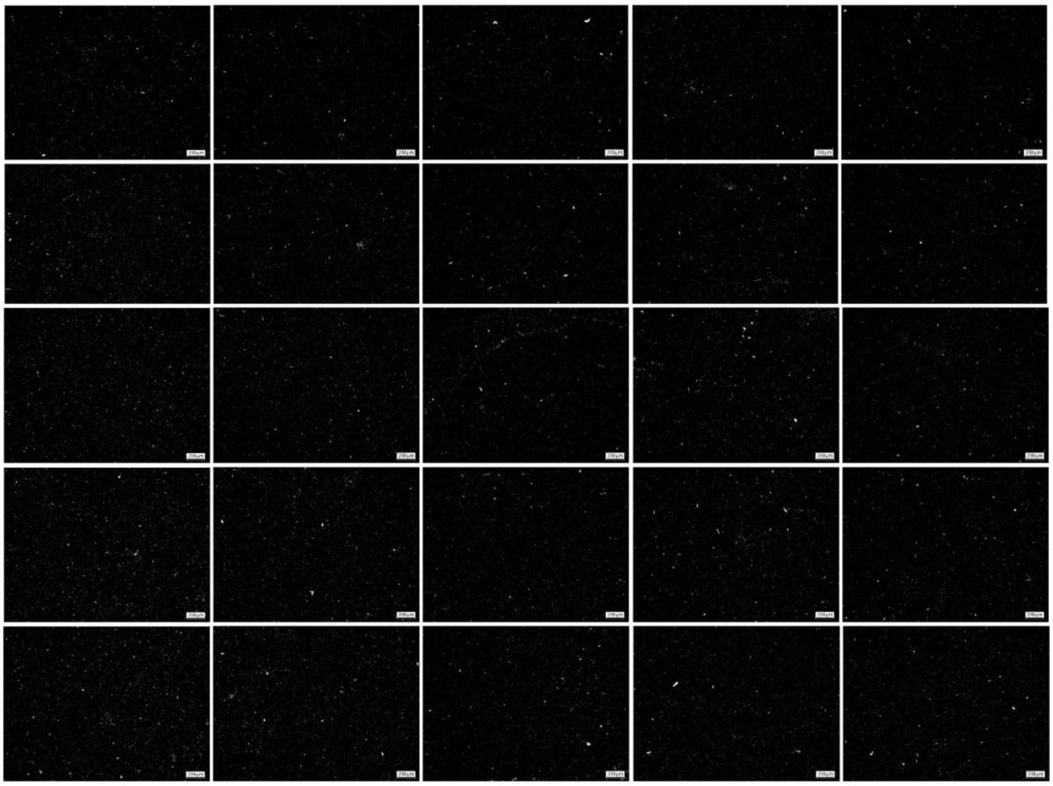

Figure S4. Array of SEM micrographs showing the PMMA plate coated with microparticles (MP_surf) after rinsing. These images were used to calculate the coating coverage values presented in Figure 4.

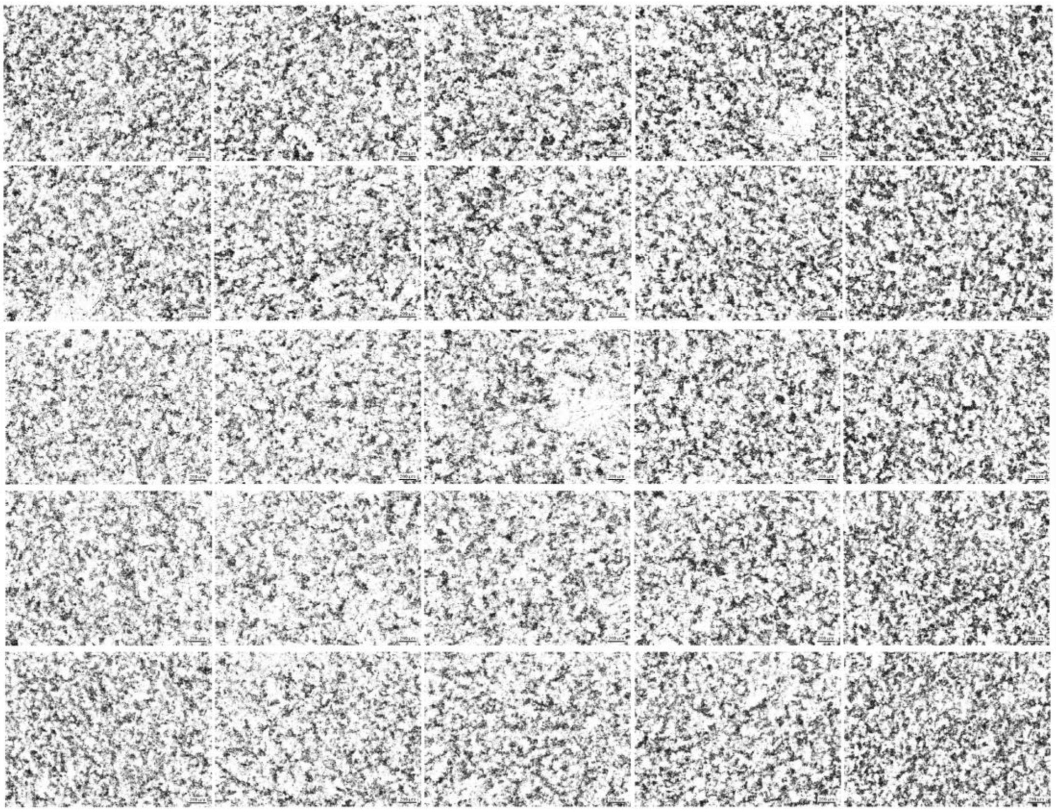

Figure S5. Array of SEM micrographs showing the PMMA plate coated with nanoparticles (NP_surf) prior to rinsing. These images were used to calculate the coating coverage values presented in Figure 4.

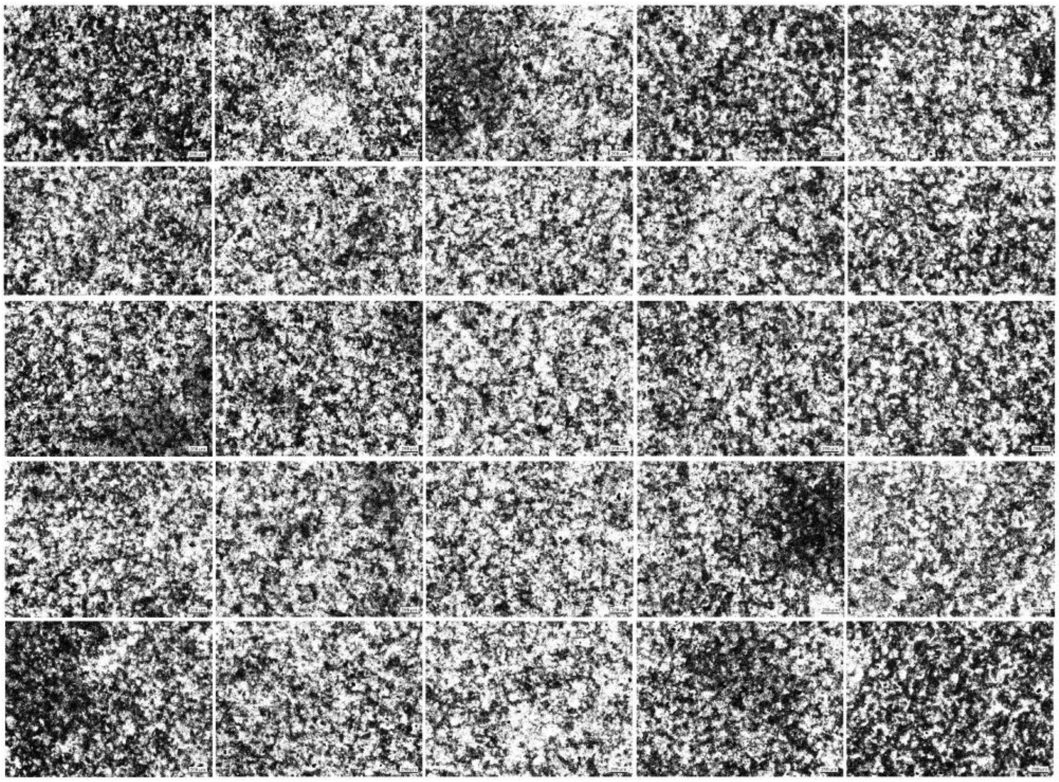

Figure S6. Array of SEM micrographs showing the PMMA plate coated with nanoparticles (NP_surf) after rinsing. These images were used to calculate the coating coverage values presented in Figure 4.
